# Exploring what patients with musculoskeletal conditions want from first point-of-contact health practitioners

**DOI:** 10.1093/rap/rkz048

**Published:** 2020-01-07

**Authors:** Jo Erwin, Kenneth Chance-Larsen, Michael Backhouse, Anthony D Woolf

**Affiliations:** 1 Bone and Joint Research Group, Royal Cornwall Hospital, Truro; 2 School of Sport and Health Sciences, University of Central Lancashire, Preston; 3 NIHR Leeds Musculoskeletal Biomedical Research Unit, University of Leeds, Leeds, UK

**Keywords:** musculoskeletal, first-contact health practitioners, national framework, patients, qualitative

## Abstract

**Objectives:**

This research was conducted to support the development of the Musculoskeletal (MSK) Health Capabilities Framework to ensure that the framework reflected patients’ priorities. The aim of this study was to explore what patients with MSK problems want from their initial consultation with a first contact health practitioner and, from the patient perspective, what characterizes a good first contact health practitioner.

**Methods:**

Focus groups were held in four locations across England. Sixteen participants, aged 19–75 years and with a self-declared MSK condition, took part (11 female, five male). Participants discussed the questions they want answered when first going to see a health professional about an MSK problem and how they would describe a good first contact health provider.

**Results:**

Participants wanted answers to questions about the nature of the problem, the management of the problem, where to get information and support to help themselves, what activities they can do and what the future holds. Values and behaviours they expect and value from first contact health practitioners include good communication skills, appreciation of impact, a willingness to discuss alternative and complementary therapies, shared decision-making and an awareness of their own limitations and when to refer.

**Conclusion:**

The MSK core capabilities framework for first contact health practitioners aims to ensure a person-centred approach in the first stages of managing any MSK problem with which a person may present. The focus groups enabled the developers of the framework to achieve a greater understanding of patient priorities, expectations and needs and allowed the patient perspective to be included in this national framework.

**Key messages**

- Findings from the study enabled the patient perspective to be included in the Musculoskeletal Health Capabilities Framework.
- The varied nature of musculoskeletal conditions and patients’ diverse care journeys make identification of priorities challenging.
- Patients want to be supported to manage and make informed decisions about their own health.

## Introduction

Musculoskeletal (MSK) conditions are the single biggest contributor to the growing burden of disability in the UK [1]. MSK conditions have an enormous impact on the quality of life for millions of patients. They account for >100 million general practitioner (GP) appointments each year and cost the National Health Service (NHS) nearly £5 billion per year [2]. Although most NHS patients with an MSK problem will initially present to their GP, they may present to a wide array of health professionals working in the community, including nurses, podiatrists, physiotherapists, occupational therapists and others. Most of these professionals are not specialists in MSK health. For people presenting with undiagnosed MSK conditions to receive appropriate care, first contact health practitioners need to have the necessary knowledge, skills and behaviour to deliver a service that meets their needs. Before 2018, in England, there was no framework outlining the competencies required by first contact health practitioners to work with patients with MSK conditions. Therefore, Health Education England and NHS England Medical Directorate commissioned the development of an MSK core capabilities framework in order to support the transformation of services, placing skilled MSK practitioners earlier in patient pathways. From its inception, the framework focused on the workforce capability to support shared decision-making, care and planning, in addition to prevention, self-management, fitness for work and meaningful behaviour change. Patient-centred care is at the heart of the framework and, as such, it is consistent with the Person-Centred Approaches framework, which was published in 2017 [3].

The MSK core capabilities framework was developed using a modified Delphi consensus approach, combining existing literature with the views of a heterogeneous, multi-professional group of expert clinicians and patients to identify core capabilities and behaviours needed by first contact practitioners [4]. In addition, the project management group sought to include the voice of non-expert patients in the framework. This article describes this process and its outcomes. It also adds to the existing United Kingdom (UK) research exploring what patients with MSK problems want from their GPs and other first contact practitioners, in addition to their expectations of GPs in relationship to MSK health [5–9]. The aims were to explore what patients with MSK problems want from their initial consultation with a first contact health practitioner and to explore with patients what characterizes a good first contact health practitioner.

## Methods

### Participants

Focus groups were held in four locations across England: Truro, Preston, Leeds and London. Participants were recruited through local patient support groups, public patient involvement groups, postings on social media sites relating to MSK health and advertising in local media. Participants were aged ≥18 years, with a self-declared MSK condition (current or within the last 1 year). Sixteen participants aged 19–75 years took part; 11 female and five male. Their conditions included inflammatory arthritis (IA), OA, back and neck pain and sports injuries.

### Data collection

The focus groups were facilitated by researchers (J.E. and K.C.-L.) and held for ∼1 h. With the participants’ agreement, the focus group discussions were digitally recorded and subsequently transcribed verbatim. The transcriptions were anonymized. J.E. is a social scientist with a background in public health and K.C.-L. is a senior lecturer in physiotherapy. Both facilitators adopted a non-judgemental, open, listening approach, being aware of the possible power dynamics that can come into play with patient focus groups. They emphasized their reliance on and appreciation of the participants’ knowledge about the phenomena under study and on their willingness to share. J.E. emphasized to the groups that she is not a medical professional, encouraging participants to be open about their views.

Focus groups were conducted with the aid of a predetermined topic guide, which was developed after a narrative review of the literature, and informal discussions with clinicians and patients (see Table 1).

**Table 1:** Patient focus group topic guide

**Introduction** There are a number of different people you can go and see if you have a musculoskeletal problem; for instance, you might go and see your general practitioner (GP), your practice nurse, a physiotherapist, an occupational therapist, a podiatrist, an osteopath, a chiropractor or a pharmacist. In this project, we are interested in finding out what questions people want answered when they first go to see someone about a musculoskeletal problem. So, we’re interested in what you want from so-called first contact providers, such as GPs and others who work in your community, rather than the hospital doctors, physiotherapists or occupational therapists that your GP might refer you to. Could you please take a minute to think about a musculoskeletal problem that you had in the past year that you went to see a doctor, nurse or other health professional about (remember, we are not including hospital visits here). Question 1. What made you decide to seek health care for your musculoskeletal problem? Question 2. What questions did you want answered when you very first went to see a health professional about this musculoskeletal problem? Question 3. How about if you had to have a follow-up appointment? How did the questions differ? Question 4. What affects how satisfied you feel with a consultation? Question 5. How would you describe a good first contact health-care provider?

### Analysis

The focus group transcripts were imported into NVivo v.11 (QSR International, Melbourne, Victoria, Australia) and analysed using deductive thematic analysis by a researcher (J.E.) [10]. All transcripts were initially reviewed and coded. Through an iterative process of reviewing transcripts and generating codes, the codes were further refined, grouped into concepts where similarities amongst codes existed, and key themes developed. One of the transcripts was independently coded by another researcher (M.B.) to check the degree of agreement between the two coders and to highlight alternative interpretations. The number of coders was restricted to two because of study time restraints. The two researchers were in general agreement in the coding of each specific unit, and there were no significant areas of disagreement. Despite the different conditions and the different patient journeys taken by participants, the key messages in relationship to what patients with MSK problems want from their initial consultation with a first contact health practitioner and what characterizes a good first point-of-contact health practitioner were very much shared. The analysts felt confident that data saturation, defined as the degree to which new data repeat what has been expressed in previous data, was achieved.

The project complies with the Declaration of Helsinki. This research project was approved by the University of Leeds Ethics panel, ethics reference number MREC 16-009. Written informed consent was obtained from the focus group participants.

## Results

It soon became apparent from the focus groups that the variation in the patients’ journey according to the duration of MSK problem (i.e. acute, a recurring problem or long term) meant that neatly dividing questions into the first visit and the follow-up visit, as was initially intended, did not match well with the patients’ experience. Participants with long-term conditions suggested that each episode of recurrence or exacerbation could be seen as a first visit, e.g. a person with long-term low back pain would need to re-access the health system for subsequent exacerbations. Therefore, the questions they might have and their expectations of their first contact health practitioner are informed by previous consultations, experiences and the nature of their condition. Consequently, in the analysis, we have not categorized responses by first and follow-up visit but have looked more broadly at the key concerns of people in the first stages of presenting with an MSK problem. These concerns can be grouped into five key themes, which are described below.

### Questions that patients want answered

#### The nature of the problem

When they go to see a first contact health practitioner about their MSK problem, participants wanted to know what was wrong and the cause of their problem. However, they did not expect all first contact providers to be able to answer these questions. In three of the four focus groups, participants expressed doubt that a GP would be able to answer these questions. Some thought it best to see a physiotherapist, who was seen as an experts in MSK problems, or, for those with IA, a rheumatologist.

Female 4: GPs are limited, that’s why they are in general practice. They have a little bit of knowledge about an awful lot of things but not specialized or in depth.

Male 4: … not to mean any offense to any GPs, but it’s like my back isn’t his speciality I think, so I wasn’t expecting him to know the answers. … So I did want to know if he could tell me there and then, kind of thing, what it was. But he couldn’t at the time, so I also sort of waited for the physio to ask because that’s more of their thing.

Most participants agreed that they would want to know what to expect and what the prognosis was for their condition.

Female 11: I mean, I like to know what the other symptoms are, how long they’re going to last, whether they’re a bacterial thing or something. Whatever the symptoms are, however long they’ll be around for, like medication, can they get rid of it. Just, like, what I’ll do. Just want to feel like I know what’s going on.

However, some participants who had long-term IA questioned whether they would ask the first contact provider about their prognosis. They felt that this was a more appropriate question for a rheumatology specialist.

Male 3: Where they [questions about prognosis] probably do come in is with a first appointment with a specialist, but I don’t think you’d ask those at your GP … because you wouldn’t expect GPs to have that knowledge.

#### The management of the problem

Patients wanted to know from their first contact health practitioner what they could do to help them with their problem.

Male 3: Yes, what can you do for me? … What can you do about my problem?…

Patients wanted to know what treatment is the most appropriate. Some participants with IA did not expect first contact health practitioners (both GPs and physiotherapists were mentioned) to be able to answer this question.

Female 4 (person with IA): I think some of these questions would be more suited for the consultant level, certainly about treatments. GPs, as I say, I do talk to mine, and we talk about the biologic drugs, but I probably have more knowledge than she does about exactly what they do and what they target. So, I think the GPs wouldn’t be able to answer some of those questions because they don’t have that in-depth knowledge.

How to manage pain and pain medication was an important issue for participants and was discussed in three of the four groups. It was recognized that the pain cannot always be eliminated, but how to reduce it to bearable levels was a key question. For some participants, the question they wanted answered was how to manage pain without resorting to ‘strong’ medications. They didn’t want to be ‘palmed off’ with painkillers and wanted to know which management options they have beyond medication and painkillers.

F6: I don’t want to have lots and lots of drugs constantly, so I may well have a little bit to un-inflame and I might have a bit of painkiller, but I don’t want the doctor to say, ‘have drugs, go home’; I want to get better properly.

Patients also wanted to know which management options they have beyond medication and painkillers.

Male 2: Is there any way you can sort of give me some exercise to reduce the pain? Not to get rid of it, that’s an impossibility, but something to reduce the pain.

Female 1: If I did see a GP about my hip, I would want to learn about it, new hip or yoga? Not just painkillers. …

A question that all the focus groups said was important was, ‘What can I do to help myself?’. There was a recognition that the participants had to take control of their condition, and they wanted to be given support to do this.

Female 1: … what can I do to alleviate it? … To be able to be in charge of the condition and it not be in charge of you, that’s my first priority.

Female 7: … I wanted something that I felt like I could be in control of, so with a physio, you know that you’re going to go and you’re going to get exercises that are going to target the particular problem, so that’s what I guess I was looking for specifically. Yeah, to be able to manage it a bit better myself.…

#### Where can I get information and support to help myself?

Participants wanted first contact health practitioners to answer their questions about where they could get information, especially self-help resources and support.

Female 8: Yes, I think related to asking about what I can do to help myself—exercises, eating and whatever—is asking for some information on where I can find stuff about self-help … it would be good if the GP or whoever could tell you about that.

Female 6: I suppose the other thing I would ask is, where can I get support if I need it? Are there any groups locally that I can get in touch with?

#### What activities can I do?

Participants wanted to know whether and when they could do the things they needed or wanted to do; this included work. For some with acute or recurrent problems, this was a key reason for going to see the first contact provider:

Male 4: I went, and my main two issues at the time were am I still ok to work? Like I wasn’t doing any massive exercise, but I was standing up for like 3–8 hours a day. I could be standing up constantly, so I was worried if pain would come from that.

However, some participants found first contact health practitioners unable to answer those questions.

Female 8: My husband is a dairy farmer. He can’t just leave the cows and it’s difficult to get help, so of course his first question to the GP was, when can I get back to work? But to be honest, he [GP] couldn’t answer that question.

Patients with long-term conditions were concerned about their legal rights in relationship to employment but felt this was beyond the remit of GPs.

Male 3: Am I able to work? Yes, that’s something a lot of people are unsure about. What your legal rights? And your employer, how much does he value you and what is he prepared to put up with? … I don’t think that’s a question that can be answered directly by the doctor. Obviously, he’s not going to counsel you on anything like your legal rights.

Other activities that participants wanted to know whether or not they could do and when were sports, going to the gym, activities of daily living, such as maintaining personal hygiene, and being able to drive.

Female 1: I get a lot of pain across the shoulder. Doing things like washing your hair … functions that are very important to being alive, what I can do?

#### The future

Participants were keen to know how they were progressing and what the future might bring. They talked about the importance of continuity of care and of being able to build a relationship with their health-care provider in order to facilitate an answer to the important questions: Have I made an improvement? And what else can be done?

Female 2: You go back and then you tell them, and you have this dialogue. It is working, my pain’s less, I can move my arm more. So this progression, this dialogue, this relationship of seeing the same person when you go back, is so important.

Male 2: … if you go back and it’s totally different people and you have to reiterate what you said the first time and go through it all over again. If you’ve tried, you want to know, have I done it properly? Can you see an improvement? And they could show you where you’ve gone wrong.

### What makes a good first contact practitioner

Participants were also asked what makes a good first contact health-care provider. They came up with a range of skills, knowledge and attitudes, which are described in the six themes below.

#### Good communication skills

Participants spoke at length of the importance of first contact health practitioners being able to listen, to put people at ease and to make the patient feel comfortable to ask questions.

Female 11: Bedside manner is really important: warm, friendly, open, listening. That’s what you need, not to feel like a number.

Male 5: Yeah, it’s a big one, listening. They really need to be able to take on board what you’re saying … You want to feel you can talk to them like your closest friends sort of thing. Obviously, you’ll never get to that point, but you’ll want to feel as comfortable.

#### Appreciation of impact

Participants emphasized the importance of health-care providers being empathic and able to appreciate the impact of the MSK condition on their lives. This was particularly true for those with long-term conditions.

Male 1: … it’s the approach. The fact they should have some sort of appreciation of how debilitating it [IA] can be on normal existence.

#### Being supportive of patients

Participants spoke of the importance of health-care providers being supportive of patients and reinforcing their feelings of self-efficacy.

Female 7: With exercises, you’ve got to find time to do them, which isn’t always easy. Perhaps the only thing I would suggest is more encouraging you and motivating you.… Believing in you, so that you can believe in yourself.

#### A willingness to discuss alternative and complementary therapies

For some of the participants, it was important that the first contact provider was open to recognizing and discussing the patient’s use of alternative and complementary medicines.

Female 11: The alternative route, herbal, doctors don’t like that. I’m on medication for other things, and if I say, ‘Can I take Echinacea, will that affect my medication?’, the doctor says, ‘Don’t ask me; I’m a doctor, not a herbalist.’ That’s not helpful when you are trying to make a sensible decision.

Female 6: I feel that doctors dismiss alternative options here. It’s such a different picture in XX; they advised me to go to yoga, looked at all the options. I think that’s very good if they can do that.

#### Shared decision-making

Participants felt that first contact health-care providers should be willing and able to learn from their patients, especially those with long-term conditions. They should be open to working with patients to make decisions and to provide the patients with sufficient and accessible information to help them in this process.

Female 2: … so the idea of responsibility of yourself … we have dual responsibility; you’re the doctor, I’m the patient. I come and I know a bit more than I used to. You know sometimes more, sometimes less.

Female 8: Be person centred; if recommending something, explain what it is and why.

#### Awareness of their own limitations and when to refer

Following on from this, participants felt it was important that first contact health practitioners have sufficient knowledge to be able to know their own limitations and when to refer on. When health-care providers do not know the answer, they should be honest and open about it.

Male 1: It would be nice if they get someone who doesn’t know exactly what they are up against to admit that they weren’t sure, however, get them [the patient] in the right place instead of just fumbling away in the dark. … to say I don’t know that, but I’ll find out.

Male 5: It’s if they have a baseline knowledge, enough to know roughly the problem and who I should refer you to. As long as they get the next stage right and they are referring you to the right people, giving you the right advice about what you should be doing ….

## Discussion

This research was conducted to support the development of the MSK Health Capabilities Framework in order to ensure that the framework reflected patients’ priorities. It explored what questions patients want answered when they first seek help for their MSK condition and the values and behaviours they expect and value from a first contact health practitioner.

Identifying what questions patients with MSK health problems want answered in the early phases of their condition is challenging because of the varied nature of MSK conditions and of the patients’ diverse journeys to care. Some conditions may be acute, such as a sports injury, some may be recurrent, such as back pain, and others may be long-term progressive conditions, such as RA. Depending on the condition and the nature of their patient journey, patients may have different expectations of their first contact health practitioner informed by previous experience. Patients agreed with the majority of the patient questions posited in the draft framework, but in addition wanted answers to questions about how they could manage their problem practically and advice on where to obtain information and support.

Patients want first contact health practitioners to be knowledgeable, to be honest and open about when they don’t know the answer and to seek expert advice, including referral, if the problem is not within their capabilities. Evidence suggests that getting the right destination for MSK referrals can be challenging for GPs, with a lack of clarity over whether patients are best sent to physiotherapy, orthopaedics, rheumatology or elsewhere [11]. There is also evidence that some GPs are unclear what allied health professionals, such as physiotherapists and occupational therapists, have to offer [12].

In terms of professional behaviours and values, patients emphasized the importance of the practitioner having the ability and inclination to communicate well with the patient, taking the time to explain and discuss options. They also emphasized the importance of continuity of care and of being treated holistically as an individual, supported and motivated to make the most of their capabilities. A willingness to engage in shared decision-making was valued. This supports National Institute for Health and Care Excellence (NICE) guidelines [13] and the emphasis placed on shared decision-making by NHS England [14, 15].

The findings from the focus groups reinforce the strong messages appearing in other professional frameworks [16–18] that health and social care professionals must work collaboratively with people who use health and community services. For those with IA and other long-term conditions, there was a wish for the consultation to be a meeting between experts, where patients and clinicians can learn from each other. They want to be supported to develop the knowledge, skills and confidence they need more effectively to manage and make informed decisions about their own health. This echoes the four principles of person-centred care put forward by the Health Foundation [19].

There are several strengths of our study. The participants in the focus groups included a range of people with differing conditions, health-care experience and age. Participants came from locations across England and were recruited through a wide variety of methods. J.E., the lead facilitator, emphasized to the groups that she is not a medical professional, encouraging participants to be open about their views even if critical of these professionals. In addition, we have explored an area that to date appears to be have been little researched. A limitation was that the majority of participants had long-term conditions, meaning that the views of those with acute conditions were less well represented. In addition, no young women (<25 years of age) took part in the groups. We are unsure why we were unable to recruit any young women to the study. A speculative explanation for this disparity is the possibility that, given that young men are more likely to take part in sports and physical activities than young women [20], they are more aware of MSK health and therefore more interested in taking part in a study. Future work must ensure that the voices of young women are heard.

### Conclusion

The MSK core capabilities framework for first point-of-contact clinicians aims to ensure a person-centred approach in the first stages of managing any MSK problem with which a person might present. For this to be achieved, first contact health practitioners must meet the expectations and needs of people with an MSK problem, address the concerns they have and foster shared decision-making. The focus groups enabled the developers of the framework to achieve a greater understanding of patient priorities, expectations and needs when first contacting a health practitioner about an MSK problem and allowed the patient perspective to be included in this national framework.

*Funding:* This work received support from the NIHR Leeds Biomedical Research Centre and PiRAtes (mid-Cornwall NRAS support group), who helped to recruit participants for the focus groups. Health Education England and NHS England commissioned the development of the framework and the associated qualitative research described in this article.

*Disclosure statement:* The authors have declared no conflicts of interest. The views and opinions expressed herein are those of the authors and do not necessarily reflect those of National Institute for Health Research, NHS or the Department of Health.
